# The Prenylflavonoid ENDF1 Overrules Central Nervous System Growth Inhibitors and Facilitates Regeneration of DRG Neurons

**DOI:** 10.3389/fncel.2019.00332

**Published:** 2019-07-24

**Authors:** Lara Bieler, Michael Vogl, Michael Kirchinger, Corinna Urmann, Herbert Riepl, Christine Bandtlow, Lars Klimaschewski, Ludwig Aigner, Sebastien Couillard-Despres

**Affiliations:** ^1^Institute of Experimental Neuroregeneration, Paracelsus Medical University, Salzburg, Austria; ^2^Spinal Cord Injury and Tissue Regeneration Center Salzburg (SCI-TReCS), Salzburg, Austria; ^3^Organic-Analytical Chemistry, Weihenstephan-Triesdorf University of Applied Sciences, Straubing, Germany; ^4^TUM Campus Straubing, Straubing, Germany; ^5^Division of Neurobiochemistry, Innsbruck Medical University, Innsbruck, Austria; ^6^Department of Anatomy, Histology and Embryology, Division of Neuroanatomy, Medical University of Innsbruck, Innsbruck, Austria; ^7^Institute of Molecular Regenerative Medicine, Paracelsus Medical University, Salzburg, Austria; ^8^Austrian Cluster for Tissue Regeneration, Vienna, Austria

**Keywords:** flavonoids, dorsal root ganglions, DRG neurons, axonal outgrowth, semaphorin, ephrin, CSPGs, neuroregeneration

## Abstract

Restoration of neuronal connectivity after lesion of the central nervous system, such as spinal cord injury, is one of the biggest challenges in modern medicine. In particular, the accumulation of axon growth inhibitory factors at the site of injury constitutes a major obstacle to structural and thus functional repair. We previously investigated a group of prenylflavonoids derived from hops for their capacity to promote neuroregeneration. We identified a molecule called ENDF1 that was very potent to enhance regrowth and branching of neurites from dorsal root ganglion neurons in culture on growth promoting substrates. In the present study, we investigated ENDF1’s capacity to promote regeneration of rat dorsal root ganglion neurons *in vitro* in the presence of three main components of the extracellular matrix acting as axon growth inhibitors: Semaphorin 3A, Ephrin A4 and mixed chondroitin sulfate proteoglycans. We report that ENDF1 application significantly promoted the percentages of sensory neurons able to regrow their neurites regardless of the presence of those inhibitors, and this to an extent similar to the one obtained after NGF treatment. Moreover, ENDF1 strongly enhanced the total neurite length and the complexity of neurites extending from neurons challenged with axon growth inhibitors. Although the impact of NGF and ENDF1 on the regeneration of neurons was similar, the activity of ENDF1 was not mediated by signaling through the TrkA receptor, indicating that each molecule act through different signaling pathways. In addition, ENDF1 did not decrease the phosphorylation of cofilin, a downstream effector of the regeneration-associated RhoA/ROCK signaling pathway. Hence, ENDF1 is a potent pro-neuroregenerative factors that could help in identifying new efficient targets for regenerative therapies of the nervous system.

## Introduction

Many therapeutic interventions currently applied following spinal cord injury aim for a stabilization of the lesion and for a limitation of exacerbating secondary damages. These interventions, however, cannot re-establish lost neuronal connectivity which would be crucial for functional regeneration. Unfortunately, in the injured central nervous system (CNS), the accumulation of axon growth inhibitors at the lesion site severely hinders regeneration. Hence, strategies supporting axonal regeneration in the lesioned CNS are strongly needed.

The extracellular matrix (ECM) is the most abundant structure found in the microenvironment of the CNS. It is mainly composed of proteins (e.g., collagens, laminins) and carbohydrate-enriched molecules named proteoglycans (Kwok et al., 2014). The chondroitin sulfate proteoglycans (CSPGs) constitute the main proteoglycans subclass occurring in the CNS. CSPGs can be subdivided into lecticans (namely aggrecan, brevican, neurocan and versican), phosphacan, small leucine-rich proteoglycans (decorin and biglycan), NG2 and neuroglycan-C (Ruoslahti, 1996; Iozzo, 1998; Kwok et al., 2014). CSPGs are synthesized by neurons, as well as glia (Crespo et al., 2007). After traumatic injury, astrocytes, microglia and various NG2 expressing cells migrate to the lesion site and contribute to the glial scar by upregulating the secretion of ECM components such as CSPGs (Galtrey and Fawcett, 2007; Goritz et al., 2011). The scar formation is on the one hand considered to be beneficial to circumscribe and prevent spreading of the lesion in the acute phase after injury. However, it is thereafter thought to hinder the axonal regeneration in the chronic phase after CNS injury (Quraishe et al., 2018).

Proteins of the semaphorin and ephrin families play crucial roles in the development of the nervous system and have system-stabilizing properties in adult organisms under physiological conditions (Dick et al., 2013; Boyd et al., 2014). However, following CNS injury, semaphorin 3A expression is elevated in the vicinity of the lesion, especially within scarred tissue (Pasterkamp et al., 2001). Additionally, ephrin A4 receptors (ephA4) also become rapidly upregulated at the lesion site, promoting astrogliosis and loss of neurite regrowth (Fabes et al., 2006; Frugier et al., 2012). Both semaphorins and ephrins are major chemorepellent axon guidance molecules present in the ECM and their accumulation at the lesion site provokes the collapse of growth cone from regenerating axons (Rohm et al., 2000; Lisabeth et al., 2013).

The anti-regenerative impact of the ECM growth inhibitors following spinal cord injury has been substantiated by studies attempting to remove or neutralize their signaling. Hence, the local application of chondroitinase ABC, degrading the CSPGs at the injury site, allowed axons to better regrow and enhanced functional recovery in animal models of spinal cord injury and stroke (Bradbury et al., 2002; Sorg et al., 2016; Wiersma et al., 2017). Kaneko et al. (2006) also reported that inhibition of semaphorin 3A by selective inhibitors considerably increased functional recovery in a rat model of spinal cord transection. Furthermore, mice deficient in ephA4 or mice treated with ephA4 inhibitors showed a remarkable reduction of astrogliosis after spinal cord injury, concomitant with a stronger axonal regrowth across the lesion site (Goldshmit et al., 2004, 2011). Therefore, the neutralization of the axon growth inhibitory activities of ECM components appears more and more as a promising strategy to improve regeneration of the injured CNS.

The flavonoid family comprises a large variety of molecules, some of which have been investigated for their therapeutic activities in disorders of the nervous system. For example, a flavonoid-rich food uptake has been suggested to prevent age-dependent cognitive decline (Gildawie et al., 2018). Moreover, flavonoids have been reported to reduce oxidative stress and amyloid-β-protein production in animal models of Alzheimer’s disease and to reduce neuronal death in models of Parkinson’s disease (reviewed in de Andrade Teles et al., 2018). We previously reported on the neuroregenerative activities of a prenylflavonoid, named ENDF1 (Enhancement of Neuronal Differentiation Factor 1) isolated from hops. ENDF1 was found to promote neurite regrowth of dorsal root ganglion (DRG) neurons *in vitro*, and in addition to promote neuronal differentiation and neuroprotection (Oberbauer et al., 2013). In the present study, we investigated the capacity of ENDF1 to promote regeneration of rat DRG neurons in the presence of ECM axonal growth inhibitors known to accumulate at the CNS lesion sites.

## Materials and Methods

### Primary Sensory Neuron Cultures

Experiments were performed in accordance to the guidelines of the “Directive 2010/63/EU of the European Parliament and of the Council of 22 September 2010 on the protection of animals used for scientific purposes.” According to the European Directive 2010/63/EU Article 3 and the Austrian legislation for experiments on living animals Tierversuchsgesetz 2012 §2c, no additional approval was required for the killing of animals with the aim of collecting tissues. DRGs for this study were isolated from postnatal day 2 (P2) Fisher-344 rats. Preparation of dorsal root ganglion neurons was done according to a protocol previously published (Haller et al., 2007). In short, pups were decapitated and DRGs were harvested along the complete spinal cord. Dissected DRGs were maintained in ice-cold DMEM medium containing 4.5 g/L D-glucose (Biochrom, Berlin, Germany) until all DRGs were harvested. Thereafter, DRGs were triturated and incubated in 5000 U/mL collagenase Type I (Life Technologies, Carlsbad, Germany) dissolved in HBSS solution containing 1.4 mM calcium chloride (Gibco, Thermo fisher Scientific, Austria) for 15 min at 37°C. DRGs were further digested at 37°C for 15 min with Accutase (Pan-Biotech, Aidenbach, Germany). Following three washes in DMEM supplemented with 4.5 g/L D-glucose, DRG neurons were resuspended in DMEM containing 4.5 g/L D-glucose, 100 U/mL penicillin, 100 μg/mL streptomycin (Pan-Biotech, Aidenbach, Germany), B27 supplement (Life Technologies, Karlsbad, Germany) and further triturated twice with fire-polished glass pipettes. Approximately 500 neurons contained in 50 μL of culture medium were seeded on the coated coverslips (see below) placed in 24 wells/plates and allowed to adhere for 30 min before adjusting the final volume of culture medium to 1 mL per well. After 16 h cells of culture, DRG neurons were fixed with 0.1 M phosphate-buffered 4% paraformaldehyde pH 7.4 for 10 min at room temperature and the regeneration of DRG neurons in the various culture conditions was analyzed.

### Determination of Inhibitor and ENDF1 Concentrations for *in vitro* Assays

Coverslips were coated using various concentrations of each inhibitors to determine the concentrations of inhibitors lowering to approximately 30% the proportion of rat DRG neurons capable of regrowing their processes within 16 h. To this end, HCl-etched glass coverslips (12 mm) were first coated with 100 μg/mL poly-L-ornithine hydrobromide (Sigma-Aldrich, MO, United States) overnight at 37°C. Afterward, coverslips were washed three times with distilled water and allowed to air dry. Human semaphorin 3A-Fc (Biomedica, Vienna, Austria) and human ephrin A4-Fc (Biomedica, Vienna, Austria) were pre-clustered 1:1 with anti-hFc-antibody (Biomedica, Vienna, Austria) for 1 h on ice. Coverslips were then coated with semaphorin 3A and ephrin A4 at concentrations of 1, 5, and 10 μg/mL, whereas chicken CSPGs (Millipore, Darmstadt, Germany) were applied at concentrations of 1, 5, and 50 μg/mL and incubated overnight at 4°C. Finally, inhibitors were removed and the coverslips were coated with 5 μg/mL laminin (Sigma-Aldrich, MO, United States) for 2 h at 37°C. Coverslips coated with 100 μg/mL poly-L-ornithine directly followed by 5 μg/mL laminin served as no-inhibitor control (Baumer et al., 2014).

The most effective ENDF1 concentration for regeneration was determined with a dose response assay. Rat DRG neurons were seeded on 5 μg/mL CSPGs-coated coverslips and treated with 1 μM ENDF1, 5 μM ENDF1, 10 μM ENDF1, 20 μM ENDF1, or 50 μM ENDF1 prepared from a 100 mM ENDF1 stock solution in DMSO. The final concentration of DMSO was adjusted to 0.05% in every well. Culture medium and medium containing 0.05% DMSO (vehicle) served as negative controls. NGF has been extensively studied for its axonal growth promoting activity on DRG neurons in culture and therefore 20 ng/mL NGF (Life Technologies, Carlsbad, Germany) in 0.05% DMSO was used as positive control for regeneration.

Further assays were performed with 10 μM ENDF1 prepared from a stock solution of 100 mM ENDF1 in DMSO. The standard vehicle control conditions corresponded to 0.01% DMSO in the culture medium and NGF 20 ng/mL (Life Technologies, Carlsbad, Germany) was used as positive control for regeneration of DRG neurons.

For the inhibition of TrkA signaling, 1 μM of the selective TrkA-receptor inhibitor (GW441756, stock solution 50 mM in DMSO, Selleck Chemicals, Munich, Germany) was added to the cultures 30 min before adding ENDF1 or NGF.

### Immunocytochemistry

Immunocytochemistry was conducted as described previously (Couillard-Despres et al., 2008). Labeling of the DRG neurons was performed using a rabbit anti-β-III-tubulin primary antibody (Abcam, #ab18207, dilution 1:1000) followed by a donkey anti-rabbit Alexa 568 secondary antibody (Invitrogen, Dilution 1:1000). Nuclear counterstaining was obtained using 1 μg/mL 2-Diamidinphenylindol (DAPI). Micrographs were acquired using a microscope for epifluorescence (Olympus IX81) and the cellSens Dimension Software (Olympus).

### PC-12 Cell Culture

PC-12 cell line (ATCC CRL-1721) was cultured in RPMI 1640 medium containing L-glutamine (Gibco, Thermofisher, Austria), 5% fetal bovine serum (Gibco, Thermo fisher scientific, Austria), 10% horse serum (Gibco, Thermo fisher scientific, Austria), 100 U/mL penicillin, 100 μg/mL streptomycin (Pan-Biotech, Aidenbach, Germany) at 37°C and 5% CO_2_ in a humidified incubator. Culture flasks were coated with 250 μg/mL poly-L-ornithine hydrobromide (Sigma-Aldrich, MO, United States). Cultures were passaged using Accutase (Pan-Biotech, Aidenbach, Germany).

For the analysis of TrkA phosphorylation and RhoA/ROCK signaling, PC-12 cells were seeded in 6-well plates (3 × 10^6^ cells/well) for 24 h. To investigate TrkA phosphorylation, PC-12 cells were treated with 10 μM ENDF1 for 5, 15, and 30 min. Treatment with 100 ng/mL NGF (Life Technologies, Carlsbad, Germany) served as positive control to induce TrkA phosphorylation (Kaplan et al., 1991). Culture medium and 0.01% DMSO were used as negative controls. To investigate impact of ENDF1 on the RhoA/ROCK signaling, PC12 cells were treated with 10 μM ENDF1 for 30 and 120 min. The selective Rho kinase inhibitor Y27632 (stock solution 50 mM in DMSO; Selleck Chemicals, Munich, Germany) was applied at 25 μM for 30 and 120 min as positive control of RhoA/ROCK signaling inhibition. Culture medium and 0.05% DMSO served as negative controls.

### Protein Extraction and Western Blotting

For the analysis of TrkA and cofilin phosphorylation, PC-12 cells were detached using a cell scraper and collected in ice-cold PBS (Biochrom GmbH, Berlin, Germany). After centrifugation at 300 × *g* and 4°C, pellets were resuspended and homogenized in 200 μL of ice-cold RIPA-buffer (Abcam, Cambridge, United Kingdom) supplemented with protease inhibitors (cOmplete, Roche, Switzerland) and phosphatase inhibitors (PhosSTOP, Roche, Switzerland). Suspensions were centrifuged at 14 000 × *g* for 20 min at 4°C and supernatants were stored at −80°C until further use. Protein concentration was determined using the Pierce BCA Protein Assay Kit (Thermo fisher scientific, Austria).

For western blotting, 10 μg of proteins per well were loaded on 4–20% TGX stain-free gels (Bio-Rad Laboratories, Austria). After electrophoresis, gels were UV-irradiated for 2.5 min using a ChemiDoc Imaging system (Bio-Rad Laboratories, Austria) according to manufacturers’ manual. Thereby, trihalo compounds in the gel react with the proteins and generated fluorophores allow for total protein loading normalization. Proteins were transferred on a 0.2 μm PVDF membrane (Bio-Rad Laboratories, Austria) using a *Trans*-blot turbo transfer system (Bio-Rad Laboratories, Austria). Membranes were incubated in blocking solution containing 0.1 M Tris (pH 7.5), 0.15 M NaCl, 5% bovine serum albumin (Sigma-Aldrich, Germany) and 0.1% Tween-20 for 1 h at room temperature. Afterward, membranes were incubated overnight at 4°C with primary antibodies diluted in blocking solution: rabbit anti-p-TrkA (#9141; 1:1000; Cell Signaling Technologies, Germany), rabbit anti-cofilin (D3F9; 1:1000; Cell Signaling Technologies, Germany), rabbit anti p-cofilin (77G2; 1:1000; Cells Signaling Technologies, Germany). Membrane were washed 3 × 10 min in TBST (Tris-buffered saline containing 0.1 M Tris (pH 7.5), 0.15 M NaCl and 0.1% Tween-20) and incubated with secondary antibody diluted in blocking solution for 1 h at room temperature: goat anti-rabbit–HRP (1:50000; Bio-Rad Laboratories, Austria). Afterward, membranes were washed 3 × 10 min with TBST and chemiluminescent signal was obtained using Clarity Western ECL Substrate (Bio-Rad laboratories, Austria) and detected with the ChemiDoc Imaging system.

### Documentation and Statistical Analyses

Experiments were performed in three independent biological replicates. Immunodetection of β-III-tubulin was used to label DRG neurons for analysis. The frequency of neurite outgrowth was assessed in a minimum of 200 β-III-tubulin-labeled DRG neurons for each condition in three biological replicates. A neurite of at least one soma size length was used as criterion to define regenerating DRG neurons. Total neurite length of neurons examined was determined by manual tracing of all β-III-tubulin-labeled neurites and branching points using Fiji (Schindelin et al., 2012). Total neurite length and the number of branching points were calculated in at least 50 neurons per condition and biological replicate. Western blots were analyzed using the Image Lab 5.2 software (Bio-Rad Laboratories, Austria). Band intensities were normalized to the total protein in the lane and to the medium controls.

Data are presented as mean value ± standard deviation. Statistical analysis was performed by one-way analysis of variance (ANOVA) with a Bonferroni Multiple Comparison *post hoc* test or student’s *t*-test using Prism7 (GraphPad, San Diego, United States). A *p*-value of ≤0.05 was considered statistically significant.

## Results

### Dose-Dependent Regeneration of DRG Neurons Seeded on ECM Inhibitors

**FIGURE 1:**
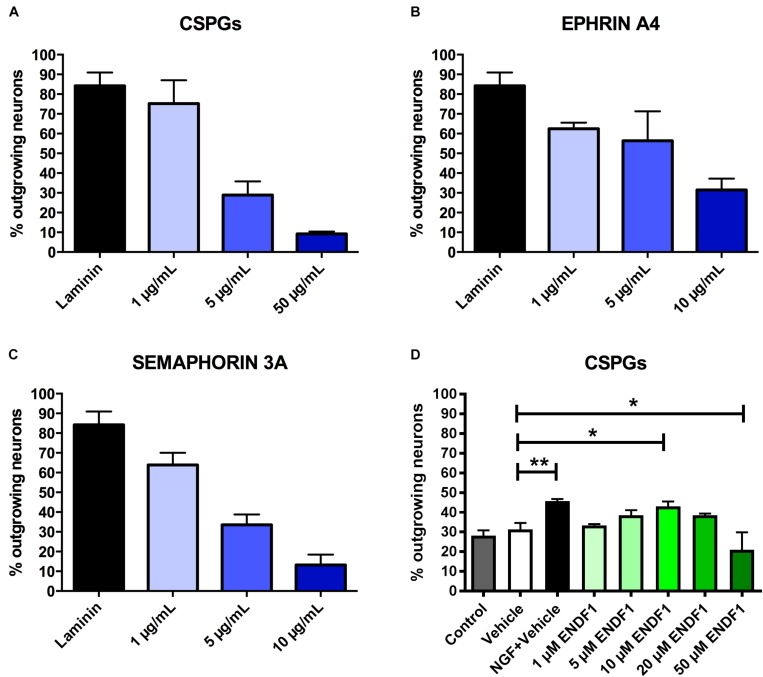
Determination of effective ECM inhibitor and ENDF1 concentrations. Different concentrations of CSPGs **(A)**, ephrin A4 **(B)** and semaphorin 3A **(C)** were coated on the coverslips to determine the optimal inhibitor concentration for the analysis of DRG neurons neurite regrowth. **(D)** ENDF1 dose-response curve for DRG neurons neurite regrowth on 5 μg/mL CSPGs. Values are shown as mean ± SD. ^*^p ≤ 0.05 and ^∗∗^*p* ≤ 0.01.

Dose-response curves for three preparations of ECM inhibitors, namely mixed CSPGs, ephrin A4, and semaphorin 3A, were established to determine the concentrations inhibiting neuronal outgrowth to the extent that only approximately 30% of the DRG neurons can spontaneously regrow neurites. This inhibition level is optimal to study neurite growth promoting substances as it allows a certain degree of spontaneous neurite regrowth and can accommodate for batch-to-batch fluctuation in inhibitory activity of the commercial inhibitor preparations. We evaluated the ability of DRG neurons to regrow their neurites on coverslips coated with 1 to 50 μg/mL CSPGs, or coated with ephrin A4 or semaphorin 3A in concentrations ranging from 1 to 10 μg/mL. The percentage of process-bearing DRG neurons was determined for each condition. As shown in Figure 1A, 28.9 ± 6.9% of DRG neurons displayed neurites 16 h after seeding on 5 μg/mL CSPGs. Similarly, 10 μg/mL ephrin A4 (Figure 1B) or 5 μg/mL semaphorin 3A (Figure 1C) limited the percentages of seeded DRG neurons growing their neurites to 31.4 ± 5.8% and 33.5 ± 5.3%, respectively. These concentrations of inhibitors were further used for this study. Neurons seeded on laminin without inhibitor displayed spontaneous neurites regrowth in a frequency of 84.2 ± 6.7%.

A dose response experiment was performed to define the most effective concentration of ENDF1 for the regeneration of DRG neurons seeded on 5 μg/mL CSPGs (Figure 1D). The highest level of DRG neurons regeneration was observed using 10 μM ENDF1 which lead to a significantly increased of the percentage of neurons with neurites (43.1 ± 2.4%), compared to cultures with 0.05% DMSO vehicle (31.3 ± 3.3%; *p* = 0.02). Higher concentrations of ENDF1 did not provide stronger regenerative activity, or even decrease the frequency of DRG neurons regenerating their neurites. Regeneration of DRG neurons exposed to 0.05% DMSO did not significantly differ from the regeneration observed in culture medium control (*p* = 0.90).

### ENDF1 Enables Neurite Regrowth Despite ECM Inhibitors

We previously reported that application of 10 μM ENDF1 in the culture medium promotes neurite regeneration of embryonic chicken DRG neurons seeded on laminin, which is a growth-supporting substrate (Oberbauer et al., 2013). In this study, we further addressed the potency of ENDF1 to promote regeneration of DRG neurons seeded on ECM inhibitors-coated coverslips (i.e., 5 μg/mL CSPGs, 10 μg/mL ephrin A4, or 5 μg/mL semaphorin 3A). The pro-regenerative activity of ENDF1 in the presence of each inhibitor was assessed by comparison to the vehicle conditions, and was in addition compared to the regenerative activity of NGF, a growth factor promoting regeneration of neurons in culture (Nusser et al., 2002).

**FIGURE 2:**
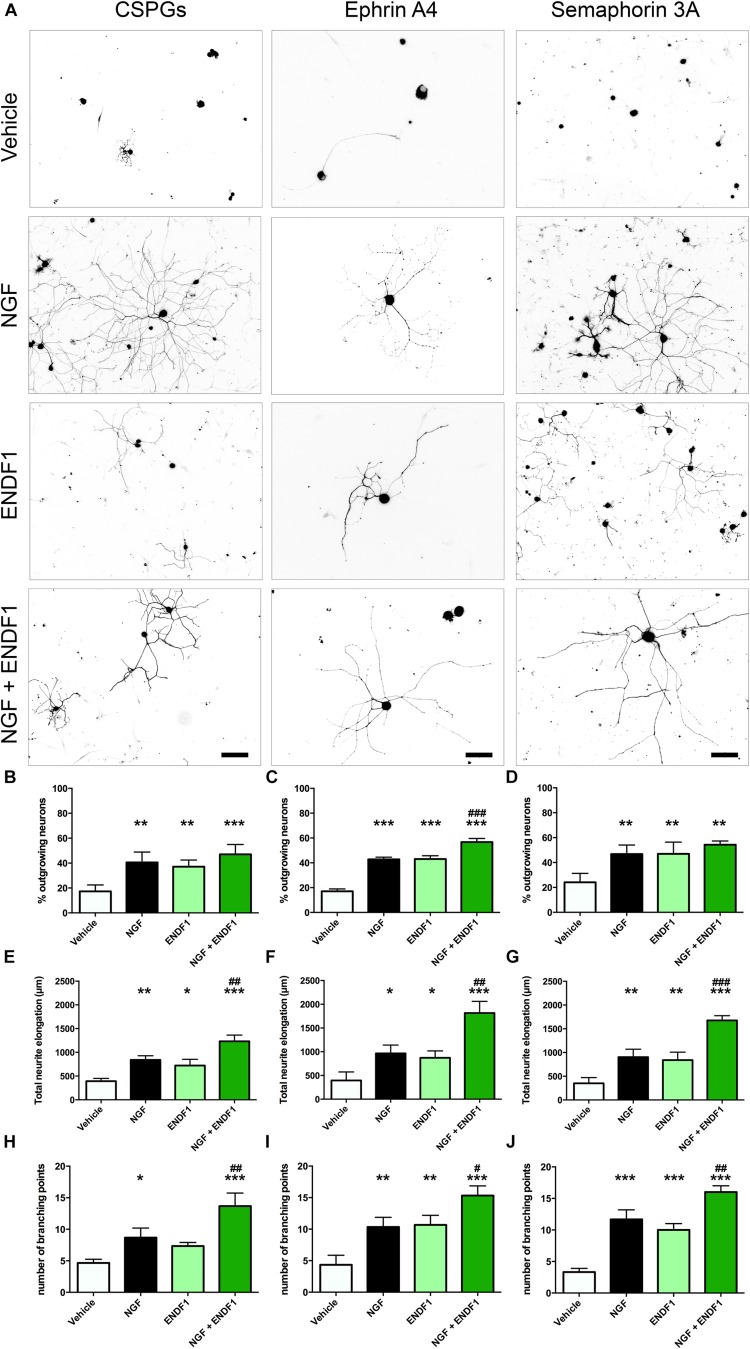
Neurite outgrowth, length and complexity in inhibitory conditions and ENDF1 treatment. **(A)** Representative micrographs showing sensory DRG neurons seeded over CSPGs, ephrin A4 and semaphorin 3A. The various cultures were treated with either vehicle, 20 ng/mL NGF, 10 μM ENDF1 or combined 20 ng/mL NGF/10 μM ENDF1. Panels **(B–D)** shows the percentage of neurons displaying neurites regrowth in the different inhibitory conditions. The total neurite length of DRG neurons regrowing their neurites in the various inhibitory conditions is displayed in panels **(E–G)**. The complexity of the regrowing neurites was evaluated by determination of the number of branching points present in the neurite arborization in the various inhibitory conditions **(H–J)**. All values are displayed as mean ± SD. ^*^p ≤ 0.05, ^∗∗^*p* ≤ 0.01, and ^∗∗∗^*p* ≤ 0.001 compared to vehicle control. ^#^*p* ≤ 0.05, ^##^*p* ≤ 0.01, and ^###^*p* ≤ 0.001 compared to NGF treatment.

When DRG neurons were seeded on CSPGs in the presence of vehicle (DMSO 0.01%), only 17.3 ± 5.2% of the neurons displayed neurites. In the presence of 10 μM ENDF1, 37.1 ± 5.4% of the DRG neurons regrow their neurites, which was a highly significant increase compared to the vehicle condition (*p* = 0.0024). Similarly, 20 ng/mL NGF highly significantly increased the percentage of neurons displaying neurites (40.6 ± 8.3%; *p* = 0.0013). The combination of both treatments, ENDF1 and NGF, was also successful to highly significantly raise the percentage of neurons developing neurites (47.0 ± 7.9%; *p* = 0.0002) as compared to the vehicle, however, the treatment with NGF and ENDF1 in combination had no additional benefit compared to the NGF treatment alone (*p* = 0.7454) (Figures 2A,B).

Furthermore, ENDF1 also counteracted the inhibitory effect of ephrin A4 in the culture. When only the vehicle was applied, 17.1 ± 1.8% of the neurons showed neurites. A treatment with ENDF1 highly significantly raised the percentage of regrowing neurons to 43.0 ± 2.7% (*p* < 0.0001). Similarly, NGF highly significantly increased the percentage of process-bearing neurons to 42.8 ± 1.8% (*p* < 0.0001). The combination of ENDF1 and NGF treatments significantly raised the percentage of DRG neurons regrowing neurites to 56.7 ± 2.9% compared to NGF solely treated neurons (*p* = 0.0003) (Figures 2A,C).

Finally, in the presence of semaphorin 3A on the coverslips, treatment with 10 μM ENDF1 raised the number of regrowing neurons from 24.2 ± 7.2% (vehicle) to 47.0 ± 9.4% (ENDF1; *p* = 0.0021). A comparable regeneration was seen upon treatment with NGF, resulting in 46.7 ± 7.3% of the neurons regrowing their neurites (*p* = 0.0013). A combined treatment with ENDF1 and NGF also significantly increased the percentage of neurons regrowing their neurites to 54.3 ± 3.0% (*p* = 0.0002). The combination of both treatments, however, had no additional benefits when compared to the application of NGF alone (*p* = 0.4003) (Figures 2A,D).

### ENDF1 Enhances Neurite Length Despite ECM Inhibitors

The extent of neurite elongation was determined by measuring the total length of neurites per neuron for each condition investigated. In the presence of CSPGs, neurites reached an average total length of 392.3 ± 58.2 μm in vehicle conditions. A treatment with ENDF1 more than doubled the neurite length, for a total length of 872.3 ± 145.7 μm (*p* = 0.0158). NGF also significantly increased the neurite length of DRG neurons regrowing on CSPGs to 840.3 ± 87.1 μm (*p* = 0.0026). A combination of ENDF1 and NGF further increased the neurite length to 1232.0 ± 132.1 μm (*p* < 0.0001 as compared to vehicle). Furthermore, the treatment with both substances together was significantly more effective on DRG neurons as compared to treatment with NGF only (*p* = 0.0060) (Figure 2E).

Under the inhibitory influence of ephrin A4, the application of ENDF1 enhanced the total neurite length (ENDF1: 872.3 ± 145.7 μm; vehicle: 393.9 ± 180.5 μm; *p* = 0.0457). A treatment with NGF also generated a significant augmentation of the neurite elongation (NGF: 965.3 ± 175.5 μm; *p* = 0.0188) and the combination of the two substances increased even more the total neurite length to 1815.0 ± 246.2 μm (*p* < 0.0001). Additionally, the treatment with NGF and ENDF1 in combination significantly enhanced neurite growth compared to NGF treatment alone (*p* = 0.0018) (Figure 2F).

In the presence of semaphorin 3A, ENDF1 treatment doubled the elongation of the neurites to a total length of 722.7 ± 130.6 μm compared to the vehicle controls (352.0 ± 119.1 μm; *p* = 0.0089). Similarly, NGF enhanced the total neurite length of DRG neurons seeded on semaphorin 3A to 902.3 ± 167.0 μm (*p* = 0.0044). The combination of ENDF1 and NGF caused a further significant increase in total neurite length to 1675.0 ± 101.4 μm (*p* < 0.0001) compared to the vehicle control. In addition, the combination of ENDF1 and NGF significantly induced more regrowth compared to the NGF alone in semaphorin 3A inhibitory condition (*p* = 0.0005) (Figure 2G).

### ENDF1 Increases Complexity of the Neurite Arborization of DRG Neurons Seeded on ECM-Inhibitors

In addition to the total neurite length, the number of branching points of the neurite arborization was quantified as an indicator of complexity for the DRG neurons regrowing their neurite in the three inhibitory conditions. When DRG neurons were seeded on CSPGs (Figure 2H), ENDF1 treatment did not significantly influence the complexity of the arborization (vehicle: 4.7 ± 0.6; ENDF1: 7.3 ± 0.6; *p* = 0.1271). In contrast, NGF treatment enhanced the number of branching points to a mean of 8.7 ± 1.5 branching points per neuron (*p* = 0.0204). The combined treatment with ENDF1 and NGF resulted in a significant increase of branching points (13.7 ± 2.1; *p* > 0.0001). Furthermore, the treatment with both substances significantly increased the number of branching points of DRG neurons seeded on CSPGs in comparison to NGF treatment alone (*p* = 0.0058).

In ephrin A4 containing cultures (Figure 2I), ENDF1 significantly increased the number of branching points to a total of 10.7 ± 1.5 per neuron compared to neurons in vehicle conditions having 4.3 ± 1.5 branching points per cell (*p* = 0.0029). NGF also significantly increased the complexity of the neurons (10.3 ± 1.5 branching points; *p* = 0.0040). A combination of ENDF1 and NGF further increased the number of branching points to 15.3 ± 1.5 (*p* < 0.0001), which is significantly higher than the average obtained with NGF treatment alone (*p* = 0.0117). Similarly, when neurite regrowth was inhibited by semaphorin 3A in the culture (Figure 2J), ENDF1 treatment significantly increased complexity of the regrowing neurites (vehicle: 3.3 ± 0.6 branching points, ENDF1: 10.0 ± 1.0 branching points, *p* = 0.0002). NGF treatment also resulted in a more complex morphology compared to vehicle conditions (NGF: 11.7 ± 1.5 branching points, *p* < 0.0001). Treatment with ENDF1 and NGF in combination further increased the number of branching points (16.0 ± 1.0) compared to NGF treatment alone (*p* = 0.0035).

### ENDF1 Signaling Does Not Require TrkA-Receptor

**FIGURE 3:**
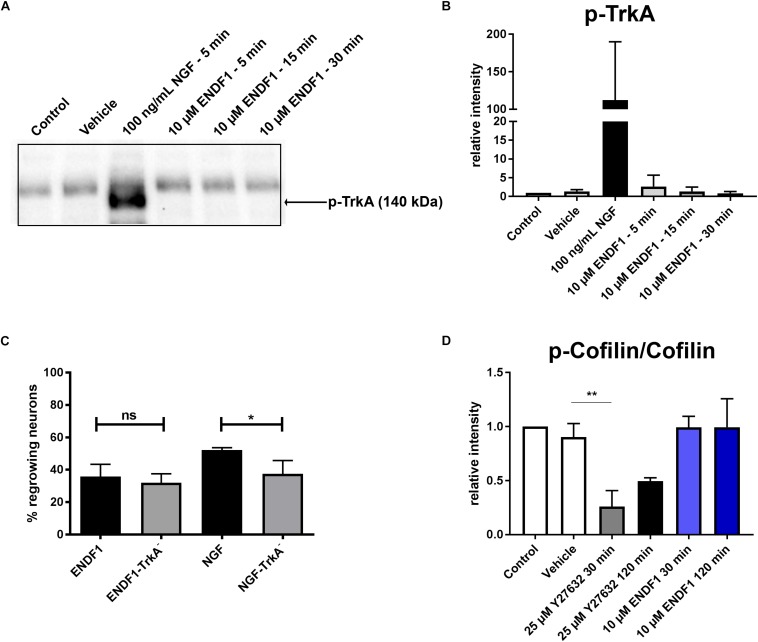
ENDF1 signaling pathways. **(A)** Western blot revealing levels of phospho-TrkA following application of 10 μM ENDF1 or 20 ng/mL NGF in PC-12 cell cultures. **(B)** Quantification of the levels of phospho-TrkA detected on the western blot shown in **(A)**. **(C)** Percentages of DRG neurons seeded on CSPGs displaying neurites after application of ENDF1 or NGF, in the presence or absence of TrkA inhibitor. **(D)** Relative levels of cofilin phosphorylation in PC-12 cell following application of ENDF1 or ROCK inhibitor (Y-27632). Values are displayed as mean ± SD. ^*^p ≤ 0.05 and ^∗∗^*p* ≤ 0.01.

Considering the similarity between the regenerative activities of ENDF1 and NGF on DRG neurons, we investigated the possibility that these two factors share a common signaling pathway. NGF mediates its pro-regenerative activities via signaling through its receptor TrkA (Zhang et al., 2000; Nusser et al., 2002). To gain insight information about a possible involvement of this NGF receptor in ENDF1 signaling, DRG neurons seeded on CSPGs were incubated with a specific TrkA inhibitor (GW441756) prior to the application of ENDF1 in the cultures. The percentage of DRG neurons regrowing their neurites in the presence or absence of TrkA inhibitor was determined after 16 h of treatment with ENDF1 or NGF. Significantly less NGF-treated DRG neurons regrew their neurites following inhibition of the NGF receptor TrkA (37.5 ± 8.2%) compared to neuron treated with NGF only (52.2 ± 1.4%; *p* = 0.0381) (Figure 3C). In contrast, the addition TrkA inhibitor only slightly decreased the percentage of ENDF1-treated DRG neurons regrowing their neurites (ENDF1 + TrkA inhibitor: 32.1 ± 5.5%; ENDF1: 35.8 ± 7.5%; *p* = 0.5198). The capacity of ENDF1 to induce TrkA phosphorylation by direct or indirect mechanisms was further investigated in PC-12 cells. Application of 100 ng/mL NGF led to a robust phosphorylation of the TrkA receptor within 5 min. On the other hand, no increase in the level of TrkA phosphorylation was detected following addition of 10 μM ENDF1 (Figures 3A,B).

### Impact of ENDF1 on RhoA/ROCK Signaling Pathway

RhoA/ROCK signaling pathway is involved in neurite outgrowth and is directly upregulated by ECM inhibitors and inhibited by pro-regenerative substances (Tonges et al., 2011). Inhibition of RhoA/ROCK signaling leads to a downregulation of LIMK-1 activity and thereby to a lower level of cofilin phosphorylation, enabling the reorganization of the actin cytoskeleton and neurite growth in PC-12 cells (Zhang et al., 2006). Application of 10 μM ENDF1 on PC-12 cells for up to 120 min did not reduce the level of cofilin phosphorylation (Figure 3D). In contrast, the addition of 25 μM Y27632, a ROCK inhibitor, reduced the level of phosphorylation of cofilin by 73.9 ± 14.7% at 30 min, as compared to the vehicle condition (*p* = 0.0028), and to a lesser extent at 120 min.

## Discussion

Restoration of neuronal connectivity rather than compensatory recovery constitutes a goal following CNS injuries, such as spinal cord injury or traumatic brain injury. Based on its pro-regenerative activity on neurite outgrowth observed *in vitro*, ENDF1 constitutes an attractive candidate to address neuronal injury *in vivo*. In the CNS, however, the accumulation of ECM components at the lesion site leads to the inhibition of axonal regrowth and currently represents a main obstacle for functional recovery after injuries. In this study, we demonstrated that ENDF1 is potent to induce neurite regrowth of DRG neurons despite the presence of inhibitory ECM molecules. Using a widespread DRG neuron culture model, we showed that ENDF1 enhanced the percentages of neurons capable of neurite regrowth, as well as the total length and complexity of their arborization despite the presence of three major ECM axon growth inhibitors, namely CSPGs, semaphorin 3A or ephrin A4. Additionally, we showed that ENDF1 is not acting via direct or indirect TrkA signaling.

The ECM-inhibitors used in this study act via their specific receptors to induce growth cone collapse. An interaction of ENDF1 with plexin-neuropilin1 complexes, Eph4A as well as PTPσ, i.e., the respective receptors of the ECM inhibitors used for this study, is very unlikely. Hence, a common downstream signaling component shared by the different inhibitors is a more plausible target for ENDF1. The RhoA/ROCK pathway is such a component relevant for neurite elongation that gets activated by the three inhibitors used in this study (Tonges et al., 2011). RhoA/ROCK inhibition induces a downregulation of LIMK-1 activity, which is leading to cofilin dephosphorylation and onset of neurite growth (Zhang et al., 2006). Previous studies have reported the modulation of Rho GTPases’ through the flavonoids isoquercetin and luteolin (Hendriks et al., 2004; Palazzolo et al., 2012). In our study, the level of cofilin phosphorylation was not decreased following incubation with ENDF1, arguing against an inhibitory activity of the later on the RhoA/ROCK signaling pathway.

Thus, the signaling pathway(s) and molecular interaction(s) conveying the regenerative activity of ENDF1 needs to be further elucidated and gives space for speculations. Among potential mechanisms, a regulation of NF-κB signaling could be involved. Several members of flavonoids subgroup named chalcones, which contained also xanthohumol and ENDF1, have been shown to inhibit NF-κB activation and signaling (Yadav et al., 2011). Lowering the activity of NF-κB has been previously proposed to improve CNS regeneration after injury (Engelmann et al., 2014). The stabilization of cytoskeletal elements could also be part of ENDF1 activity supporting neurite regeneration. For example, the microtubule stabilizing agent taxol can significantly increase axonal regeneration after optic nerve injury (Sengottuvel et al., 2011). Similarly, the application of another microtubule-stabilizing compound named epothilone B augmented the length and number of axons showing regenerative growth at the lesion site and accelerated the locomotor recovery in rodent models of spinal cord injury (Ruschel et al., 2015). Since the capacity of some flavonoids to stabilize microtubules has been previously reported in the context of anti-cancer drug development, the same mechanisms could apply in ENDF1 regenerative modes of action on neurites (Touil et al., 2009; Mukhtar et al., 2015).

Stabilization of the microtubule network by ENDF1 could also be indirect. We reported that the application of ENDF1 on mouse embryonic forebrain cells strongly induced the expression of DCX, a microtubule-stabilizing protein expressed in immature neurons (Oberbauer et al., 2013). DCX belongs to a family of microtubule-binding proteins that includes DCLK1 and DCLK2 which, in contrast to DCX, are also expressed in mature neurons of the CNS. In 2015, Nawabi et al. reported that following injury of retinal ganglion neurons, DCLK1 and DCLK2 are among the most dramatically downregulated proteins. Introducing an over-expression of DCLK2 through AAV vector following optic nerve crush resulted in a significantly better regeneration of the axons from retinal ganglion neurons (Nawabi et al., 2015). Therefore, ENDF1 could modulate the expression of genes involved in axonal regeneration, such as the member of the DCX family.

Finally, modulation of calcium channels further represents a possible mode of action of ENDF1 on axonal regeneration. Dysregulation of axonal calcium influx and storage is very detrimental in the acute phase following CNS injury (reviewed in Couillard-Despres et al., 2017). Moreover, Tedeschi et al. (2016) demonstrated that calcium channel subunits expressed in the adult CNS, such as alpha2delta2a subunit of voltage-gated calcium channels (VGCCs), are strong inhibitors of axonal regeneration. Targeted inhibition of this VGCC subunit using Pregabalin improved axonal regeneration following spinal cord injury in a mouse model. A recent study demonstrated that flavonoids can also regulate calcium channels and the hops-derived flavonoids 6-prenylnaringenin and 8-prenylnaringenin were shown to block calcium T-channels, alleviating thereby neuropathic pain after sciatic nerve ligation (Sekiguchi et al., 2018). Hence, modulation of calcium channels activity by ENDF1 should be further scrutinized as a potential target leading to improved regeneration.

In summary, the lack of axonal regrowth is currently one of the major obstacles in functional recovery after injury of the CNS. With this study, we substantiated the pro-neuroregenerative activity of the prenylated flavonoid ENDF1 for the structural repair. We demonstrated that ENDF1 strongly induced neurite regrowth and branching in DRG neurons despite the presence of axon growth inhibitors of the ECM accumulating in the lesioned CNS. Although ENDF1’s modes of action are still fairly unknown and need to be further investigated, ENDF1 constitutes a promising small molecule for regenerative therapy of the injured CNS.

## Ethics Statement

Experiments were performed in accordance with the guidelines of the “Directive 2010/63/EU of the European Parliament and of the Council of 22 September 2010 on the protection of animals used for scientific purposes.” No experiments were performed on living animals. According to the European and Autrian directives, no additional authorizations are required for the sampling of tissues on freshly sacrificed animals.

## Author Contributions

LB, MV, LA, LK, CB, HR, and SC-D contributed to the conception and design of the study. LB, MV, MK, and CU conducted and analyzed the experiments. LB and MV performed the statistical analysis. LB and SC-D wrote the manuscript. All authors contributed to manuscript revision, and read and approved the submitted version.

## Conflict of Interest Statement

LA and SC-D own a patent on the use of chroman-like cyclic prenylflavonoids for the medical intervention in neurological disorders. The remaining authors declare that the research was conducted in the absence of any commercial or financial relationships that could be construed as a potential conflict of interest.
